# Supplementary material for: Twelve Weeks of Web-Based Low to Moderate Physical Activity Breaks with Coordinative Exercises at the Workplace Increase Motor Skills but Not Motor Abilities in Office Workers—A Randomised Controlled Pilot Study
Source: Int J Environ Res Public Health. 2023 Jan 25;20(3):2193. doi: 10.3390/ijerph20032193 (PMC9915999; doi:10.3390/ijerph20032193)
Supplement: Supplementary file 1 [file ijerph-20-02193-s001.zip › Twelve weeks of _Supplementary Material Table S1_Scharf_Tilp.pdf]

Supplementary Material

Table S1. Information of the Evaluation of the intervention group (n=16).

**Question**				
	**%**	**n**	**Mean**	**SD**
**"How did you enjoy the physical activity breaks?"**				
I enjoyed them very much	81.3	13	1.25	0.58
I enjoyed them	12.5	2		
I rather enjoyed them	6.3	1		
I rather did not enjoy them	0.0	0		
I did not enjoy them	0.0	0		
**"Do you agree that the presentation of the sessions was diversified and lively?"**				
I agree	93.8	15	1.06	0.25
I rather agree	6.3	1		
I rather disagree	0.0	0		
I disagree	0.0	0		
**"Do you agree that the mobility exercises were comfortable and enjoyable for you?"**				
I agree	93.8	15	1.06	0.25
I rather agree	6.3	1		
I rather disagree	0.0	0		
I disagree	0.0	0		
**"Do you agree that the relaxation exercises were comfortable and relaxing for you?"**				
I agree	87.5	14	1.13	0.34
I rather agree	12.5	2		
I rather disagree	0.0	0		
I disagree	0.0	0		
**"The juggling exercises were _______________for me most of the time."**				
too challenging	31.3	5	1.94	0.77
neither too challenging nor not challenging	43.8	7		
too challenging as well as not challenging	25.0	4		
not challenging	0.0	0		
**"The balance exercises were _______________for me most of the time."**				
too challenging	12.5	2	2.25	0.86
neither too challenging nor not challenging	62.5	10		
too challenging as well as not challenging	12.5	2		
not challenging	12.5	2		
**"Would you agree that the participation had a positive effect on your health?"**				
I agree	68.8	11	1.44	0.81
I rather agree	25.0	4		
I rather disagree	0.0	0		
I disagree	6.3	1		

Table S1 (Continued).

Question				
	**%**	**n**	**Mean**	**SD**
**"How have you observed the positive effect on your health?"** (open ended question)				
improvement of physical well-being		5		
improvement of psychological well-being		5		
improvement of motor abilities		7		
**"Would you agree that the participation had a positive effect on your working routine?"**				
I agree	68.8	11	1.56	0.96
I rather agree	12.5	2		
I rather disagree	12.5	2		
I disagree	6.3	1		
**"How have you observed the positive effect on your health?"** (open ended question)				
improvement of concentration		5		
enhanced awareness for taking breaks		4		
adding a variety to the working routine		3		
improvement of the working structure		1		
improvement of relaxation		1		
improvement of productivity		1		
**"Would you agree that the participation has resulted in being more physical active in general?"**				
yes	62.5	10		
no	37.5	6		
**"How have you observed being more physically active ?"**				
being more physically active before or after work	30.0	3		
being mor physically active during work	30.0	3		
using the stairs instead of the elevator	20.0	2		
no answer	20.0	2		
**"Have you planned to practice the juggling exercises after your participation?"**				
yes	93.8	15		
no	6.3	1		
**"Have you planned to practice the mobilization exercises after your participation?"**				
yes	62.5	10		
no	37.5	6		
**"Have you planned to practice the balance exercises after your participation?"**				
yes	37.5	6		
no	62.5	10		
**"Have you planned to practice the relaxation exercises after your participation?"**				
yes	25.0	4		
no	75.0	12		
